# Data feedback and behavioural change intervention to improve primary care prescribing safety (EFIPPS): multicentre, three arm, cluster randomised controlled trial

**DOI:** 10.1136/bmj.i4079

**Published:** 2016-08-18

**Authors:** Bruce Guthrie, Kimberley Kavanagh, Chris Robertson, Karen Barnett, Shaun Treweek, Dennis Petrie, Lewis Ritchie, Marion Bennie

**Affiliations:** 1 Population Health Sciences Division, University of Dundee, Dundee DD2 4BF, UK; 2 Department of Mathematics and Statistics, University of Strathclyde, Glasgow, UK; 3 Centre for Population Health Sciences, University of Edinburgh, Edinburgh, UK; 4 Health Services Research Unit, University of Aberdeen, Aberdeen, UK; 5 Centre for Health Policy, University of Melbourne, Melbourne, Australia; 6 Department of Academic Primary Care, University of Aberdeen, Aberdeen, UK; 7 Strathclyde Institute of Pharmacy and Biomedical Sciences, University of Strathclyde, Glasgow, UK; 8 Information Services Division, NHS National Services Scotland, Edinburgh, UK

## Abstract

**Objective** To evaluate the effectiveness of feedback on safety of prescribing compared with moderately enhanced usual care.

**Design** Three arm, highly pragmatic cluster randomised trial.

**Setting and participants** 262/278 (94%) primary care practices in three Scottish health boards.

**Interventions** Practices were randomised to: “usual care,” consisting of emailed educational material with support for searching to identify patients (88 practices at baseline, 86 analysed); usual care plus feedback on practice’s high risk prescribing sent quarterly on five occasions (87 practices, 86 analysed); or usual care plus the same feedback incorporating a behavioural change component (87 practices, 86 analysed).

**Main outcome measures** The primary outcome was a patient level composite of six prescribing measures relating to high risk use of antipsychotics, non-steroidal anti-inflammatories, and antiplatelets. Secondary outcomes were the six individual measures. The primary analysis compared high risk prescribing in the two feedback arms against usual care at 15 months. Secondary analyses examined immediate change and change in trend of high risk prescribing associated with implementation of the intervention within each arm.

**Results** In the primary analysis, high risk prescribing as measured by the primary outcome fell from 6.0% (3332/55 896) to 5.1% (2845/55 872) in the usual care arm, compared with 5.9% (3341/56 194) to 4.6% (2587/56 478) in the feedback only arm (odds ratio 0.88 (95% confidence interval 0.80 to 0.96) compared with usual care; P=0.007) and 6.2% (3634/58 569) to 4.6% (2686/58 582) in the feedback plus behavioural change component arm (0.86 (0.78 to 0.95); P=0.002). In the pre-specified secondary analysis of change in trend within each arm, the usual care educational intervention had no effect on the existing declining trend in high risk prescribing. Both types of feedback were associated with significantly more rapid decline in high risk prescribing after the intervention compared with before.

**Conclusions** Feedback of prescribing safety data was effective at reducing high risk prescribing. The intervention would be feasible to implement at scale in contexts where electronic health records are in general use.

**Trial registration** Clinical trials NCT01602705.

## Introduction

Prescription drugs significantly improve patients’ outcomes but are also a major cause of harm in both primary and hospital care.^1^ ^2^ ^3^ Approximately one in 20 hospital admissions is caused by adverse drug events,^1^ and at least half of these adverse drug events are preventable.^4^ The classes of drug most often implicated are antiplatelet drugs (including aspirin), diuretics, non-steroidal anti-inflammatory drugs, warfarin, opioids, β blockers, angiotensin converting enzyme inhibitors/angiotensin receptor blockers, and hypoglycaemic drugs.^4^ Mortality has most frequently been associated with prescribing of non-steroidal anti-inflammatory and antiplatelet drugs,^1^ although several less commonly prescribed drugs also cause significant numbers of deaths, including antipsychotics in older people with dementia.^5^ Several consensus validated sets of indicators of potentially inappropriate or high risk primary care prescribing are available,^6^ ^7^ ^8^ ^9^ and high risk prescribing measured using these indicator sets is both common and variable between primary care practices (although not all such prescriptions will be inappropriate, as benefit will outweigh risk in at least some patients).^10^

Compared with hospital based studies, relatively few trials have looked at interventions to improve safety of prescribing in primary care, despite primary care physicians in most developed countries being responsible for prescribing most community dispensed drugs. Much previous research has focused on pharmacist led interventions, although the effect of such interventions is variable and sometimes negative, at least partly owing to lack of integration with existing primary medical care.^11^ ^12^ The PINCER trial found a statistically and clinically significant reduction in targeted high risk prescribing in a UK primary care context from implementing an intervention combining educational outreach, feedback, and pharmacist led review of patients receiving high risk prescribing.^13^ More recently, the DQIP complex intervention comprising educational outreach, informatics to identify patients and support general practitioner led review, and small financial incentives to review was shown to reduce high risk prescribing of non-steroidal anti-inflammatory and antiplatelet drugs in UK primary care by approximately one third and was associated with significant reductions in emergency hospital admissions for gastrointestinal bleeding and heart failure.^14^ However, complex interventions such as PINCER and DQIP are relatively difficult and expensive to implement at scale.

Audit and feedback is an attractive intervention from this perspective because countries and health systems are increasingly creating comprehensive patient level datasets from electronic prescribing or electronic medical record data. In this context, feedback is relatively inexpensive to implement across whole systems, potentially allowing expensive interventions to be deployed more selectively. The 2012 Cochrane review of trials of feedback found good evidence that feedback is effective, but with a wide range of observed effects across studies.^15^ Although feedback interventions targeting prescribing were in general more effective than those targeting other outcomes, only three trials included in the review examined the effect of feeding back data on safety of prescribing (two targeting benzodiazepine use^16^ ^17^ and one targeting risky non-steroidal anti-inflammatory drug use^18^). None found feedback to be effective in reducing high risk prescribing, although only one trial was judged to have low risk of bias, emphasising that the evidence base for feedback is weak in the context of prescribing safety.^15^

We aimed to add to the limited evidence about the effectiveness of feedback of data on prescribing safety and to fill gaps in the broader evidence about feedback identified by the most recent systematic review by carrying out a large trial (EFIPPS) of an intervention developed using both theory and empirical evidence to design the intervention in collaboration with clinicians and with comparison of different feedback formats, clearly defined primary outcomes, and adjustment for baseline performance.^15^ We therefore developed two theory informed formats for feedback on prescribing safety and tested their effectiveness at reducing high risk prescribing to patients compared with emailed educational material in a three arm cluster randomised trial in 262 primary care practices in Scotland.

## Methods

### Study design and participants

The study was a three group, parallel arm, pragmatic, cluster randomised controlled trial, described in more detail in the published protocol.^19^ Cluster randomisation was appropriate because feedback of high risk prescribing data was at practice level. Practices were eligible if they were located in one of three Scottish health boards (geographically defined administrative organisations in the National Health Service in Scotland), if their registered list size was at least 250 patients (all practices smaller than this are unusual in the patients served—for example, by serving only homeless people), if at least 93% of their prescriptions could be linked to individual patients, and if they were established before 1 January 2011. Practices were excluded from follow-up or analysis if they ceased to exist during the trial or if they merged with a practice from a different arm during the trial.

Decisions about individual patient care remained the sole responsibility of the practice and the individual general practitioners working in it, so consent by patients was not required. No changes to the protocol, eligibility criteria, or outcomes occurred after the trial started.

### Randomisation and blinding

Practices were randomised to each arm on a 1:1:1 basis. As all practices were randomised at the same time, balancing the allocation sequence was unnecessary, but randomisation was stratified by participating health board (three strata, as boards have a remit to ensure that general practice prescribing is effective, safe, and value for money but vary in the amount of prescribing support provided to practices) and baseline high risk prescribing as measured by the primary outcome (in thirds). The trial statistical team used a random number generator to randomise each practice to one of three groups, and the Tayside Clinical Trials Unit independently carried out random allocation of each of these groups to the three trial arms. The project team were blinded to the allocation during the conduct of the trial. Practices were not formally blinded; however, as they were not asked for consent, practice staff were not aware that they were in a trial. All outcomes were measured using routinely recorded dispensed prescribing data by using pre-defined indicators, neither of which could be influenced by practices (except by changing their prescribing) or the research team. The trial statisticians were blinded for data cleaning and analysis.

### Interventions

A detailed description of the development of the interventions and examples of study materials has been previously published.^20^ The two interventions evaluated were feedback of prescribing safety data and feedback plus a theory informed behavioural change component. All practices in each arm received the same intervention with no tailoring beyond feedback being specific to each practice, and no modifications were made during the trial.

At the start of the trial, all practices received a short educational newsletter describing the targeted high risk prescribing and recommending that practices review patients at potential risk; this was similar to existing NHS Scotland material regularly sent to practices. NHS Scotland Information Services Division sent the educational newsletter (appendix 1^20^), which included a link to a webpage with downloadable searches to facilitate identification of patients in practices’ electronic medical records and further information about the targeted prescribing. Practices in arm 1 received only the educational newsletter, which is routine practice in NHS Scotland, plus support for searching, which is not routine. The arm 1 comparator against which the two more active interventions were evaluated was therefore modestly enhanced “usual care,” chosen to reflect a simple to implement “best practice” comparator and to overcome health boards’ concerns about identifying potentially risky prescribing and doing nothing whatsoever about it.

Practices in arm 2 (feedback) were additionally sent feedback of their rate of the targeted prescribing benchmarked against the rate achieved by the 25% of Scottish practices with the most optimal rates in the year before feedback started. The choice of benchmark represents current practice in NHS Scotland to use a “best in class” comparison rather than mean or median performance. Feedback design drew on knowledge of factors associated with greater response and included both presentation of graphical data showing change over time versus the benchmark and a written explanation of why the indicators were important and what actions practices should take in response. Feedback of practices’ rates of the six individual measures of high risk prescribing was sent quarterly by email for five rounds for the year June 2012 to June 2013, with the email signed by senior staff in the health board and NHS Scotland Information Services Division. Educational material and feedback was sent to a single practice email address used by the participating health boards to communicate routinely with practices, which was either a generic practice email address (typically checked by an administrative staff member) or the address of the practice manager or a nominated prescribing lead (typically a general practitioner). The research team had no direct control over what recipients did with the email or attached educational material or feedback.

Practices in arm 3 (feedback plus theory informed behavioural change component) received the same educational newsletter and feedback, but the feedback document additionally included a one page theory informed behavioural change component intended to increase the likelihood that practices would respond to the feedback by searching for and reviewing patients receiving the targeted prescribing. We created four different behavioural change components focused on the three domains of the theory of planned behaviour (attitudes, subjective norms, and perceived behavioural control) and an action planning component based on the health action process approach.^21^ ^22^ Each of the five rounds of feedback contained one of these components, with the action planning intervention delivered twice. Appendix 2 gives examples of the behavioural change components, and appendix 3 gives an anonymised example of feedback including the action planning component (the feedback only arm had a blank page 2 but was otherwise identical).^20^

### Outcomes

We selected outcomes from measures that had been validated in one or more recent formal consensus studies,^8^ ^23^ on the basis of their perceived importance and their feasibility to implement using national prescribing data. Selection was done by the project Steering Group, which included representatives from each of the three participating health boards, NHS Scotland Information Services Division (who manage the national data), and the research team. Box 1 lists the six individual indicators selected. We measured all outcomes by using routinely available dispensed prescribing data held by NHS Scotland. Measurement was at the level of the patient, using binary variables whereby, at the end of each quarter, patients with risk factors for adverse drug effects (for example, having a prescription for an oral anticoagulant) were defined as receiving or not receiving a specified high risk prescription in that quarter (for example, prescription of an oral non-steroidal anti-inflammatory drug without gastroprotection). The primary outcome was a binary composite measuring the proportion of patients particularly at risk of an adverse event from the specified prescribing who received one or more high risk prescriptions defined by the six secondary outcomes. The outcomes pre-specified at trial registration are the same as in the published protocol,^19^ and all are reported in this paper.

**Box 1: Primary and secondary outcomes**

**Primary outcome**

Proportion of patients included in one or more of the defined six individual secondary outcomes (denominator) who receive any high risk prescription (numerator)*

**Secondary outcomes**

S1: Proportion of patients aged 75 years and over (denominator) who receive a prescription for an oral antipsychotic (numerator)†
S2: Proportion of patients aged 65 years and over and currently treated with a diuretic and an angiotensin converting enzyme inhibitor or angiotensin receptor blocker (denominator) who receive a prescription for an oral non-steroidal anti-inflammatory drug (NSAID) (numerator) (the “triple whammy”)
S3: Proportion of patients aged 75 years and over (denominator) who receive a prescription for an oral NSAID without co-prescription of a gastroprotective drug (numerator)
S4: Proportion of patients aged 65 years and over and currently treated with aspirin or clopidogrel (denominator) who receive a prescription for an oral NSAID without co-prescription of a gastroprotective drug (numerator)
S5: Proportion of patients currently treated with an oral anticoagulant (denominator) who receive a prescription for an oral NSAID without co-prescription of a gastroprotective drug (numerator)
S6: Proportion of patients currently treated with an oral anticoagulant (denominator) who receive a prescription for aspirin or clopidogrel without co-prescription of a gastroprotective drug (numerator)

*For all indicators, proportion is defined as numerator/denominator, usually multiplied by 100 to convert to percentage†As proxy of oral antipsychotic prescribing to older people with dementia. The prescribing dataset does not include diagnostic data, but our previous analysis of general practice data had shown that more than half of patients aged 75 years and over who received a prescription for an antipsychotic had a diagnosis of dementia. Given that dementia is under-recorded in this age group, our belief was that most over-75s given an antipsychotic would have dementia, and that even those without dementia would potentially benefit from having their treatment reviewed^24^

### Patient involvement

The study design drew on qualitative analysis of data from interviews with patients carried out during development of another intervention targeting safety of primary care prescribing,^25^ but there was no patient or public involvement in the study design, choice of outcome measures, or study conduct and no assessment of the burden of the intervention on patients. No patients were asked to advise on interpretation or writing up of results. There are no plans to disseminate the results of the research to study participants or the relevant patient community.

### Sample size

We estimated the required sample size by using the n4prop function in the CRTSize library in R,^26^ ^27^ with the number of patients per practice (mean 700), the primary outcome baseline rate (6.1%), and the intra-class correlation coefficient (1.26%) all calculated using the same routine dataset used to measure outcomes in the main trial. The planned primary comparisons in the study were the two intervention arms (arm 2 and arm 3) compared with usual care (arm 1) at 15 months, each to be tested at a significance level of 0.025. With 85 practices randomised to each arm, the study had 93% power to detect a 25% reduction in the percentage of high risk prescribing (from 6.1% to 4.5%) between usual care and the two intervention arms at the end of the study. We chose 25% on the basis of the findings of the PINCER intervention,^13^ for which the closest outcome (non-steroidal anti-inflammatory drug prescribing in people with a history of peptic ulcer) had a 42% reduction in odds at six months.^13^ We expected the EFIPPS intervention to be less effective as it was less intense, so we considered a 25% reduction important enough to use as the basis of the sample size calculation.

### Statistical analysis

We measured outcomes as repeated quarterly cross sections, meaning that every measurement includes all eligible patients (permanently registered and having a risk factor for adverse drug effects from the targeted prescribing at the time of measurement). The main trial analysis used a multilevel logistic regression model comparing the proportion receiving a high risk prescription in the final quarter of the study in each arm, adjusted for two variables used to stratify randomisation (health board and high risk prescribing at baseline), with the practice identifier as the clustering variable. We tested the effect of each intervention arm 15 months after the intervention was implemented separately against the usual care arm at the 2.5% significance level, to control for an overall 5% significance level. We used the R package lme4 to evaluate the model. To consider the change in trends over time, we used generalised estimating equations to construct a change point model using quarterly measurement from quarter 1 2011 to quarter 3 2013. Time was centred at baseline, scaled to yearly units, and again adjusted by the randomisation stratification variables. We then modelled the change in slope as the interaction between time and study arm in the period after intervention, which was coded as a dummy variable that took the value 0 before the intervention and 1 afterwards. We evaluated the binomial generalised estimating equations, adjusted for health board and high risk prescribing at baseline, by using an unstructured correlation structure to model the temporal correlation in the observations for each practice using the R package geepack.

## Results

The three health boards included 278 primary care practices, of which 262 (94%) were eligible for inclusion and randomised, with 170 659 registered patients particularly vulnerable to harm from the targeted prescribing at baseline. Four (1.5%) practices with 948 (0.6%) patients at risk at baseline were lost to follow-up owing to practices merging or splitting, and data from the remaining 258 practices were analysed (fig 1). Baseline characteristics of practices and patients were similar across arms. The mean age of patients at risk was 77 years, and 44% were male (table 1). Across all arms, 6.0% (95% confidence interval 5.9% to 6.2%) of patients particularly vulnerable to harm from the targeted prescribing were receiving a prescription for one or more high risk drugs at baseline, varying at individual indicator level from 0.8% (0.7% to 1.0%) for prescription of non-steroidal anti-inflammatory drugs without gastroprotection to people already receiving an oral anticoagulant to 7.8% (7.5% to 8.0%) for prescription of non-steroidal anti-inflammatory drugs to older people already receiving an angiotensin converting enzyme inhibitor/angiotensin receptor blocker and a diuretic (table 1).

**Figure f1:**
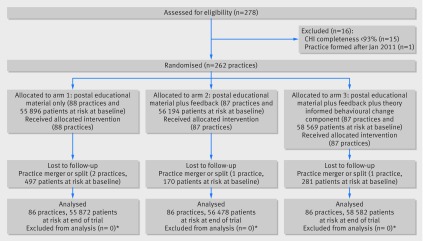
**Fig 1** Trial profile. CHI=Community Health Index (NHS Scotland unique patient identifier). *Design was repeated cross sectional analysis of routine data from all patients, so all eligible patients were analysed

**Table 1 tbl1:** Baseline characteristics. Values are numbers (percentages) unless stated otherwise

	Arm 1: education plus support for searching (n=88 practices; n=55 896 patients)	Arm 2: as arm 1 plus feedback (n=87 practices; n=56 194 patients)	Arm 3: as arm 2 plus theory informed behavioural change component (n=87 practices; n=58 569 patients)
**Practices**			
Mean (SD) list size	6360 (3005)	6538 (3290)	6893 (3388)
Training*	29 (33)	31 (36)	31 (36)
Dispensing*	3 (3)	6 (7)	0 (0)
Mean (SD) QOF achievement*	97.5 (4.2)	98.5 (3.2)	98.5 (2.7)
Median (IQR) No of patients in primary outcome indicator	584 (429)	623 (527)	600 (413)
**Patients**			
Median (IQR) age, years	77 (10)	77 (11)	77 (10)
Male sex	24 625 (44.1)	24 847 (44.2)	25 804 (44.1)
Scottish Index of Multiple Deprivation fifth:			
1 (deprived)	9966 (17.8)	10 597 (18.9)	12 102 (20.7)
2	14 056 (25.1)	15 232 (27.1)	14 204 (24.3)
3	9959 (17.8)	10 746 (19.1)	9064 (15.5)
4	7907 (14.1)	7749 (13.8)	8716 (14.9)
5 (affluent)	11 762 (21.0)	9620 (17.1)	11 907 (20.3)
Unknown	2246 (4.0)	2250 (4.0)	2576 (4.4)
Urban/rural:			
Urban	47 365 (84.7)	47 440 (84.4)	50 107 (85.6)
Rural or unknown	8531 (15.3)	8754 (15.6)	8462 (14.4)
Health board A	12 850 (23.0)	13 505 (24.1)	13 941 (23.8)
Health board B	16 093 (28.8)	17 716 (31.5)	19 239 (32.8)
Health board C	26 953 (48.2)	24 973 (44.4)	25 389 (43.3)
Mean (SD) No of drugs/patient	6.1 (3.6)	6.2 (3.7)	6.1 (3.7)
**Baseline performance**			
Primary outcome	3332/55 896 (6.0)	3341/56 194 (5.9)	3634/58 569 (6.2)
Secondary outcomes†:			
S1: aged ≥75 plus antipsychotic	691/34 427 (2.0)	697/33 575 (2.1)	741/36 113 (2.1)
S2: “triple whammy”	1187/15 632 (7.6)	1166/15 341 (7.6)	1326/16 291 (8.1)
S3: aged ≥75 plus NSAID	1016/34 427 (3.0)	1001/33 570 (3.0)	1176/36 113 (3.3)
S4: antiplatelet plus NSAID	706/27 519 (3.6)	768/28 479 (2.7)	771/28 297 (2.7)
S5: oral anticoagulant plus NSAID	55/5715 (1.0)	49/5754 (0.9)	42/5903 (0.7)
S6: oral anticoagulant plus antiplatelet	278/5715 (4.9)	277/5754 (4.8)	258/5903 (4.4)

IQR=interquartile range; NSAID=non-steroidal anti-inflammatory drug.

*Training=accredited for postgraduate general practitioner training; dispensing=dispenses as well as prescribes drugs in rural areas with no pharmacy; QOF achievement=percentage of Quality and Outcomes Framework pay for performance points achieved.

†Outcomes are defined in box 1. Benchmark rates used in feedback (those achieved by 25% of Scottish practices with most optimal prescribing in year before feedback started were S1 1.3% (median practice rate across all arms 2.2%), S2 4.8% (median 7.2%), S3 1.3% (median 2.9%), S4 1.2% (median 2.4%), S5 0% (median 0.5%), and S6 2.2% (median 4.1%).

At baseline, 6.0% of patients particularly vulnerable to harm in arm 1 (usual care) were receiving a high risk prescription, compared with 5.9% in arm 2 (feedback only) and 6.2% in arm 3 (feedback plus theory informed behavioural change component), falling to 5.1%, 4.6%, and 4.6% by 15 months (table 2). After adjustment for the two stratifying variables (health board and third of baseline high risk prescribing), the primary analysis found an odds ratio for receiving a high risk prescription of 0.88 (95% confidence interval 0.80 to 0.96; P=0.007) in arm 2 (feedback) compared with arm 1 (usual care) and of 0.86 (0.78 to 0.95; P=0.002) in arm 3 (feedback plus theory informed behavioural change component) compared with arm 1.

**Table 2 tbl2:** Prevalence of high risk prescribing at end of intervention by allocation arm, and primary analysis of intervention effect. Values are numbers (percentages) unless stated otherwise

Outcome	Arm 1: education plus support for searching (usual care)	Arm 2: as arm 1 plus feedback	Arm 3: as arm 2 plus theory informed behavioural change component	Adjusted odds ratio (95% CI)*	
				Arm 2 *v* arm 1	Arm 3 *v* arm 1
**Primary outcome**					
Receipt of any high risk prescription	2845/55 872 (5.1)	2587/56 478 (4.6)	2686/58 582 (4.6)	0.88 (0.80 to 0.96); P=0.007	0.86 (0.78 to 0.95); P=0.002
**Secondary outcomes**					
S1: aged ≥75 plus antipsychotic	661/34 533 (1.9)	641/33 824 (1.9)	676/36 282 (1.9)	1.01 (0.89 to 1.14); P=0.91	1.02 (0.90 to 1.15); P=0.76
S2: “triple whammy”	1007/15 268 (6.6)	913/14 855 (6.1)	978/15 827 (6.2)	0.91 (0.81 to 1.03); P=0.12	0.91 (0.81 to 1.02); P=0.13
S3: aged ≥75 plus NSAID	797/34 533 (2.3)	637/33 824 (1.9)	748/36 282 (2.1)	0.77 (0.65 to 0.90); P=0.001	0.82 (0.70 to 0.96); P=0.01
S4: antiplatelet plus NSAID	517/27 103 (1.9)	486/28 170 (1.7)	446/28 873 (1.5)	0.88 (0.75 to 1.04); P=0.14	0.82 (0.69 to 0.96); P=0.02
S5: oral anticoagulant plus NSAID	40/6193 (0.6)	38/6294 (0.6)	30/6537 (0.5)	0.92 (0.57 to 1.49); P=0.74	0.73 (0.44 to 1.21); P=0.22
S6: oral anticoagulant plus antiplatelet	276/6193 (4.5)	230/6294 (3.7)	202/6537 (3.1)	0.82 (0.68 to 1.00); P=0.05	0.72 (0.58 to 0.87); P<0.001

NSAID=non-steroidal anti-inflammatory drug.

*All models adjusted for two stratification variables (health board and third of primary outcome baseline performance); secondary outcome analyses additionally adjusted for baseline performance on each secondary outcome.

†Defined in box 1.

Although the study was not powered to detect differences in the six individual indicators, in the pre-specified secondary analysis examining them the estimated odds ratios for five of the indicators (S2 to S6) were similar to the primary analysis (although only statistically significant for four comparisons) (table 2). In contrast, we found no evidence of effectiveness for indicator S1 (prescription of antipsychotics in people aged 75 and over: odds ratios of 1.01 (0.89 to 1.14) for arm 2 versus arm 1 and 1.02 (0.90 to 1.15) for arm 3 versus arm 1).

In the pre-specified secondary analysis of change in trend in the primary outcome for the three arms, we found evidence that the targeted high risk prescribing was falling before the intervention (odds ratio for change per year before the intervention 0.92, 0.91 to 0.94). The segmented regression analysis estimates the immediate effect of the intervention as the odds ratio for the average level of high risk prescribing in the quarter after the intervention compared with the quarter before (after adjustment for trend) and the change in trend in high risk prescribing expressed as the odds ratio for the change in slope over the year of follow-up (fig 2 and table 3). In arm 1 (usual care), we saw no statistically significant immediate change in level of high risk prescribing or change in slope after receipt of the educational intervention. In arm 2 (feedback), no immediate change in level occurred but we saw a statistically significant and clinically important change in slope towards a steeper reduction (odds ratio per year of follow-up 0.87, 0.83 to 0.92). In arm 3 (feedback plus theory informed behavioural change component), we saw an immediate reduction in the level of high risk prescribing (odds ratio 0.96, 0.93 to 1.00) and a statistically and clinically significant change in slope towards a steeper reduction (odds ratio per year of follow-up 0.88, 0.84 to 0.93).

**Figure f2:**
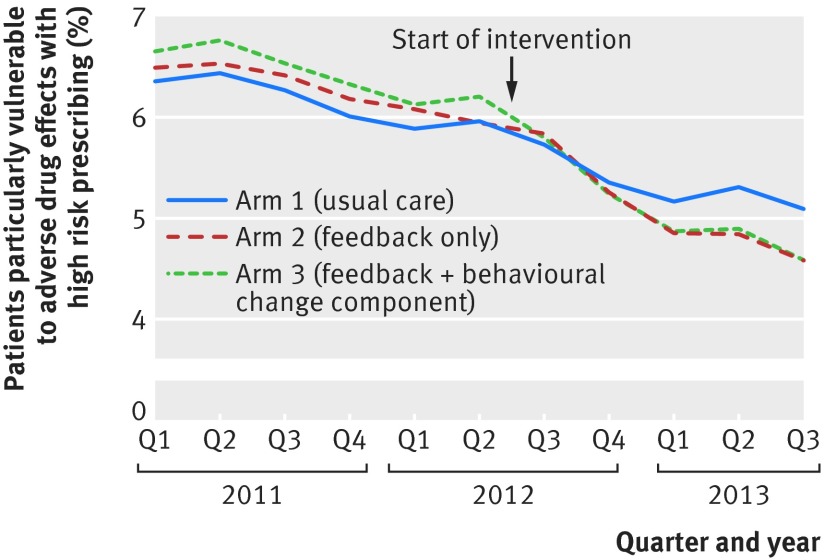
**Fig 2** Change in composite high risk prescribing in three trial arms

**Table 3 tbl3:** Immediate change in level and change in trend in composite high risk prescribing after intervention*

Outcome	Odds ratio (95% CI) per year	P value
Time trend (change per year before intervention)	0.92 (0.91 to 0.94)	<0.001
Change in level of high risk prescribing in quarter immediately after intervention started:		
Arm 1—usual care	0.97 (0.94 to 1.01)	0.10
Arm 2—feedback	1.00 (0.96 to 1.03)	0.85
Arm 3—feedback plus behavioural change component	0.96 (0.92 to 1.00)	0.05
Change in time trend per year after intervention:		
Arm 1—usual care	0.99 (0.94 to 1.04)	0.72
Arm 2—feedback	0.87 (0.83 to 0.92)	<0.001
Arm 3—feedback plus behavioural change component	0.88 (0.84 to 0.93)	<0.001

*Adjusted for two stratification variables (health board and third of primary outcome baseline performance).

## Discussion

Feedback of high risk prescribing at practice level in both formats was effective at reducing the targeted high risk prescribing compared with a simple educational intervention with support for identifying patients, with similar estimated effect sizes for both active intervention arms in the primary analysis of change from baseline (a 12% reduction on the odds of high risk prescribing in the feedback only arm 2 and a 14% reduction in the feedback plus theory informed behavioural change component arm 3). In time trends analysis, the targeted prescribing was decreasing before intervention in all three arms, possibly as a result of the cumulative effect of related regulatory risk communications in previous years.^24^ We found no evidence that the emailed educational material and online support for searching in usual care arm 1 had any significant effect, consistent with the wider evidence base showing that posted educational material is ineffective at changing practice.^28^ Both of the intervention arms were associated with a change in trend towards a faster reduction in high risk prescribing, with some evidence that the theory informed behavioural change component led to a faster initial fall (although no clinically important difference by study end). The study was not powered to detect differences in the six individual indicators, but five had estimated reductions in rates comparable to the overall finding for the composite outcome, the exception being prescription of antipsychotics in people aged 75 years and over, for which we found no evidence of any effect.

### Strengths and limitations of study

Strengths of the study included that the intervention was carefully developed using the process recommended by the Medical Research Council Complex Interventions Framework,^29^ with explicit use of theory to create two feedback formats to evaluate. We paid close attention to ensuring that the evaluated intervention was embedded in real world practice, and the trial itself involved more than 94% of primary care practices in three geographical areas.^15^ Follow-up used routinely collected data, and all eligible patients (those permanently registered and with risk factors for adverse drug effects from the targeted prescribing) were included in the analysis in all three arms. In contrast to many previous studies in this field,^15^ analysis used a pre-specified primary outcome and was adjusted for baseline performance. Analysis was limited to change in the targeted prescribing, however, and the possibility exists of unmeasured harm from stopping targeted drugs, including an increase in pain (although we would expect this to lead to further review), an increase in cardiovascular events from stopping antiplatelets (although the indications for dual antiplatelet or combined antiplatelet and anticoagulant are relatively narrow and most historical prescribing is not in the context of recent thrombotic events), or worsening behavioural disturbance if antipsychotics are stopped in people with dementia (although the only trial evidence in this context does not show harm^30^ ^31^).

A limitation of the behavioural change component was that the resources used to develop it were constrained by what the Advisory Group considered would be realistic to use in any future real world NHS implementation and by the space and delivery constraints of being embedded in the automated feedback. However, this is more a constraint of the highly pragmatic design brief, which emphasised creation of an intervention that could be implemented at scale, rather than a limitation of the study. The primary analysis did not find any evidence that the addition of the low intensity behavioural change intervention led to a clinically more important reduction in the targeted prescribing than did feedback alone (a reduction in odds of 14% versus 12%, although the trial was not powered to make this comparison). However, given what seems to be a more rapid response to feedback with the implemented behavioural change component in the secondary analysis, the additional effectiveness of more intense behavioural change interventions in this context is an important avenue for further research.

An additional limitation shared with almost all feedback studies is that whether the effect of the intervention will be sustained once feedback ceases is as yet unclear.^15^ Also, some non-steroidal anti-inflammatory drugs and aspirin can be bought over the counter without a prescription, so the targeted high risk prescribing of non-steroidal anti-inflammatory drugs and antiplatelets is likely to be underestimated both before and after the intervention. We also cannot be certain that some patients did not replace stopped prescriptions by purchasing their own drugs. Finally, whether feedback would be equally effective in reducing other types of high risk prescribing is uncertain, particularly given the lack of effect on antipsychotic prescribing in older people, which is more likely to be initiated by a specialists than is the case for non-steroidal anti-inflammatory drugs in particular and for which responsibility for review may be ambiguous.

### Comparison with other studies

Although many trials of data feedback have been conducted in other contexts, previous studies of feedback of prescribing safety data have not found feedback to be effective (two studies of a single round of feedback combined with an educational intervention targeting prescribing of benzodiazepines and non-steroidal anti-inflammatory drugs^16^ ^18^ and one study of three rounds of two monthly feedback targeting benzodiazepine prescribing^17^). In contrast, this study showed a clinically important reduction in the targeted prescribing, possibly because of the use of repeated feedback from an authoritative NHS organisation over 15 months, clear guidance on expected behaviour, and a comparison with a “best in class” benchmark that three quarters of practices were not already meeting, all of which have been shown to be associated with greater response to feedback.^15^

The observed effect size is smaller than that for two higher intensity interventions evaluated in cluster randomised trials in UK primary medical care. In the PINCER trial, the odds ratio for the three primary outcomes at six months ranged from 0.51 to 0.73, although unlike in our study the effectiveness of the intervention was waning by 12 months, with odds ratios for the three primary outcomes at 12 months ranging from 0.63 to 0.91.^13^ In the DQIP trial, the odds ratio for the primary outcome (a composite of nine measures of high risk prescribing of non-steroidal anti-inflammatory drugs and antiplatelets) was 0.63 (0.57 to 0.68), and this effect was sustained in the 12 months after the intervention ceased, reflecting that the intervention reduced general practitioners’ subsequent initiation of high risk prescribing.^14^ However, a key advantage of automated feedback interventions is that the cost of scaling delivery across entire health systems is much less than for more intensive interventions. Although targeting a different type of prescribing, a recent trial of social norm feedback to the 20% of English general practices with the highest rates of antibiotic prescribing also showed a small but significant reduction in targeted prescribing, consistent with carefully designed feedback delivered across large number of practices being a reasonable strategy with small but important effects.^32^

An alternative to this type of feedback intervention for existing prescribing is point of care reminders or alerts. The most recent Cochrane review of the effectiveness of point of care reminders estimated the median improvement in prescribing quality or safety associated with their implementation to be 3.3%, but with considerable heterogeneity across the 21 trials examining prescribing outcomes.^33^ Only three of the studies targeted prescribing safety outcomes, with effect sizes that were respectively clinically insignificant,^34^ similar to this study (10% relative improvement^35^), and somewhat larger than this study (18% relative improvement^36^). However, the considerable heterogeneity in outcomes targeted means that the relative effectiveness of point of care interventions compared with those that prompt review of existing prescribing is uncertain, although the two approaches are likely to be complementary.

### Conclusions and policy implications

This highly pragmatic trial showed the effectiveness of a low intensity feedback intervention delivered by the NHS and implemented across nearly all practices in three geographical areas. With the rapid growth of patient level datasets based on electronic medical records or pharmacy claims data, the potential for feedback interventions to improve prescribing safety is considerable, and many healthcare systems could deploy similar interventions now. In particular, given the relative ease with which feedback can be implemented at scale, it has a highly plausible place as a universal core of improvement in safety of prescribing to be supplemented by other more intensive interventions in practices that do not respond to feedback alone.^13^ ^14^

The minimum requirements for implementation are a patient level prescribing dataset to allow construction of safety indicators^6^ ^8^ ^9^ ^37^ and the ability to attribute prescribing either to a practice (which is possible in NHS Scotland) or to an individual prescriber (which will be feasible in some other contexts). Although prescribing of non-steroidal anti-inflammatory drugs and antiplatelets has strong face validity, and this study provides evidence that feedback is effective for the targeted measures, the choice of indicators for feedback should be based on what is known or believed to be particularly problematic in the local context of implementation. For example, dangerous opiate use might be judged to be a more urgent target in North America than in the UK, where opiate related deaths are much less common reflecting relatively low UK prescribing rates of oxycodone, hydrocodone, and fentanyl.^38^ ^39^ However, given the uncertainty about whether other prescribing will be equally sensitive to feedback, all implementation should aim to evaluate effectiveness carefully by using interrupted time series methods at a minimum.^40^ Understanding whether effects are sustained when feedback ceases, evaluating a range of behavioural change interventions to accompany feedback, and identifying the most effective and cost effective blend of lower and higher intensity interventions to improve safety of prescribing in wider health systems are important areas for future research.

**What is already known on this topic**

Extensive evidence shows that audit and feedback is an effective intervention across a range of healthcare contexts and outcomes with small to moderate effects on the targeted quality or safety measure
Little evidence exists for the effectiveness of feedback in the context of prescribing safety, and more generally older audit and feedback trials were often methodologically limited

**What this study adds**

This study used an intervention intended to reduce high risk prescribing, with comparison of different feedback formats (feedback only (arm 2) and feedback plus a theory informed behavioural change intervention (arm 3))
There was no evidence that a simple educational intervention with enhanced support for searching (usual care arm) significantly reduced the targeted high risk prescribing
Feedback in both formats was effective at reducing high risk prescribing compared with usual care (12% reduction in the odds of high risk prescribing in arm 2 and 14% in arm 3)

## Web Extra

Extra material supplied by the author

## Notes

We thank Jackie Caldwell, Iain Bishop, Grant Wyper, Kjersti Fergusson, and Martyn Ritchie in NHS National Services Scotland Information Services Division and all members of the Advisory Group for their contribution.

Contributors: BG had the idea for the study and co-wrote the proposal with MB and CR, with input from ST, DP, and LR. BG and MB led the development of the feedback, and KB led the development of the theory informed behavioural change component, with input from all authors. KK and CR led the statistical analysis. All authors contributed to the interpretation of the findings. BG wrote the first draft of the paper; all authors contributed to editing and approval of the final manuscript. BG is the guarantor.

Ethical approval: The study was reviewed and approved by the NHS East of Scotland Research Ethics Service (reference 11/ES/0001). As the interventions are all variants of improvement methods routinely used by the NHS and were co-designed and distributed by an NHS organisation, the Research Ethics Service did not require consent by practices. Decisions about individual patient care remained the sole responsibility of the practice and the individual general practitioners working in it, so consent by patients was also not required.

Data sharing: The data controller of the data analysed is NHS National Services Scotland. Patient level data are available subject to standard information governance requirements for use of anonymised, unconsented NHS Scotland data (http://www.informationgovernance.scot.nhs.uk/pbpphsc/home/for-applicants/). The statistical code used is available from the trial statistician Chris Robertson (chris.robertson@strath.ac.uk).

Transparency statement: The lead author affirms that this manuscript is an honest, accurate, and transparent account of the study being reported; that no important aspects of the study have been omitted; and that any discrepancies from the study as planned (and, if relevant, registered) have been explained.
